# Supplementary material for: Comparative Metabolomics of Fruits and Leaves in a Hyperdiverse Lineage Suggests Fruits Are a Key Incubator of Phytochemical Diversification
Source: Front Plant Sci. 2021 Aug 30;12:693739. doi: 10.3389/fpls.2021.693739 (PMC8435686; doi:10.3389/fpls.2021.693739)

**Supplemental data for: Comparative metabolomics of fruits and leaves in a hyperdiverse lineage suggests fruits are a key incubator of phytochemical diversification**

**Gerald F. Schneider^1,2*^, Diego Salazar^3^, Sherry B. Hildreth^1,4^, Richard F. Helm^4^, Susan R. Whitehead^1^**

^1^ Department of Biological Sciences, Virginia Polytechnic Institute and State University, Blacksburg, VA, USA

^2^Department of Biology, Utah State University, Logan, UT, USA

^3^Department of Biological Sciences, International Center for Tropical Botany, Florida International University, Miami, FL, USA

^4^ Department of Biochemistry, Fralin Life Sciences Institute, Virginia Polytechnic Institute and State University, Blacksburg, VA, USA

*** Correspondence:**Gerald F. Schneider
jerry.schneider@usu.edu

| **Table S1:** Random Forest - Boruta variable importance summary. Chemical classification is per ClassyFire as previously described. Occurrences of compounds with VIS significantly greater than shadow variables were summed for each organ across all 3 samples in all 12 species, i.e. each compound has a maximum occurrence sum of 36 for each organ. As compounds were rarely restricted to only one organ, compounds occurring in more than one organ were counted in each of the organs in which they occurred. | | | | | | |
| --- | --- | --- | --- | --- | --- | --- |
| **Chemical Class** | **m/z**  **([M+H]^+^)** | **Sum of occurrences of compounds**  **with VIS > shadow** | | | | **Variable importance score** |
|  |  | **Leaf** | **Unripe pulp** | **Ripe pulp** | **Ripe seed** |  |
| Benzene and substituted derivatives | 362.097 | 22 | 13 | 32 | 29 | 3.6 |
| Carboxylic acids and derivatives | 274.140 | 0 | 1 | 5 | 23 | 2.9 |
| Carboxylic acids and derivatives | 275.124 | 9 | 17 | 32 | 31 | 3.7 |
| Flavonoids | 311.092 | 22 | 36 | 26 | 22 | 2.3 |
| Organooxygen compounds | 281.071 | 27 | 18 | 32 | 35 | 2.4 |
| Organooxygen compounds | 288.108 | 11 | 14 | 28 | 34 | 3.6 |
| Organooxygen compounds | 319.150 | 2 | 7 | 19 | 29 | 4.8 |
| Organooxygen compounds | 323.145 | 10 | 8 | 20 | 34 | 3.5 |
| Organooxygen compounds | 343.091 | 2 | 13 | 14 | 32 | 4.0 |
| Organooxygen compounds | 466.204 | 5 | 3 | 8 | 28 | 4.6 |
| Organooxygen compounds | 467.188 | 3 | 4 | 10 | 28 | 3.2 |
| Organooxygen compounds | 522.203 | 35 | 21 | 33 | 32 | 3.7 |
| Prenol lipids | 535.271 | 35 | 19 | 21 | 11 | 3.4 |
| Unknown | 220.170 | 34 | 6 | 2 | 0 | 7.4 |
| Unknown | 269.114 | 10 | 8 | 8 | 32 | 3.6 |
| Unknown | 301.144 | 9 | 20 | 6 | 34 | 5.8 |
| Unknown | 318.300 | 0 | 26 | 16 | 36 | 6.4 |
| Unknown | 322.161 | 2 | 1 | 3 | 30 | 5.2 |
| Unknown | 439.142 | 36 | 19 | 24 | 24 | 3.8 |
| Unknown | 568.427 | 33 | 12 | 12 | 5 | 4.7 |
| Unknown | 613.483 | 33 | 12 | 11 | 8 | 5.0 |
| Unknown | 797.518 | 32 | 19 | 17 | 3 | 4.2 |
| Unknown | 954.614 | 25 | 17 | 7 | 1 | 3.0 |
| **Sum** |  | **397** | **314** | **386** | **541** |  |

**Figure S1:** Molecular network visualizations for individual species. Two network visualizations are shown for each of the 12 *Piper* species analyzed. Network visualizations are shown in alphabetical order, by species. In each pair of visualizations, the above is color-coded by chemical class, while the below is color-coded by organ-level compound occurrence. Node and edge arrangement and compound annotation are as described in “Molecular Networking” methods. Enlarged, diamond-shaped nodes represent compounds identified by the Boruta analysis as important for distinguishing among organs. Compounds are coded as occurring in “fruit” if they occur in one or more of the three sample types (unripe pulp, ripe pulp, or seeds). Compounds not present in a species are shown with high transparency node shading (chemical class visualization) or gray node shading ( organ occurrence visualization).


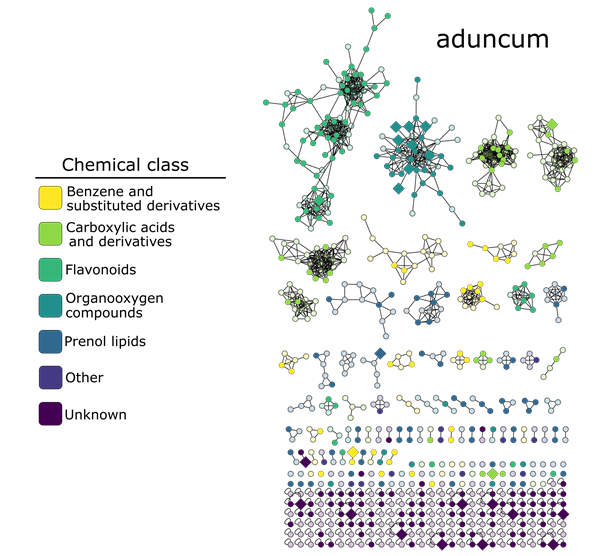


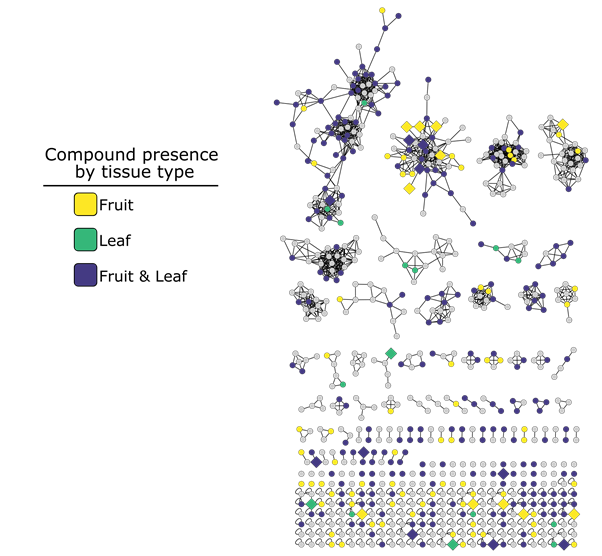


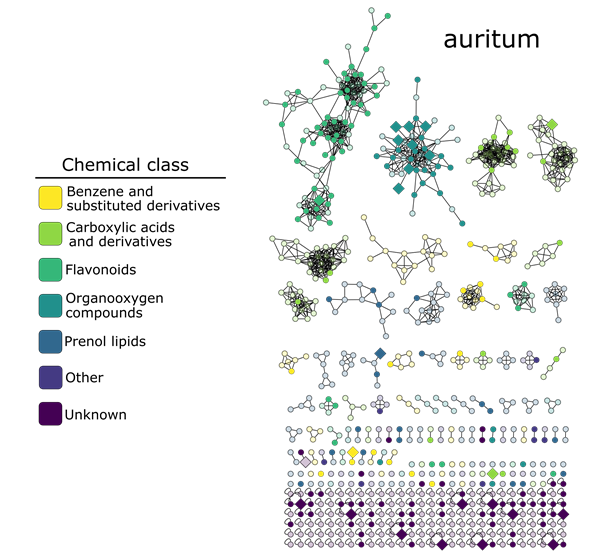


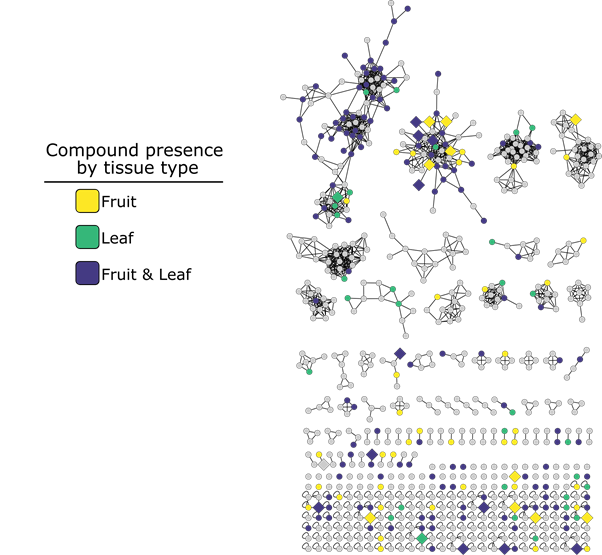


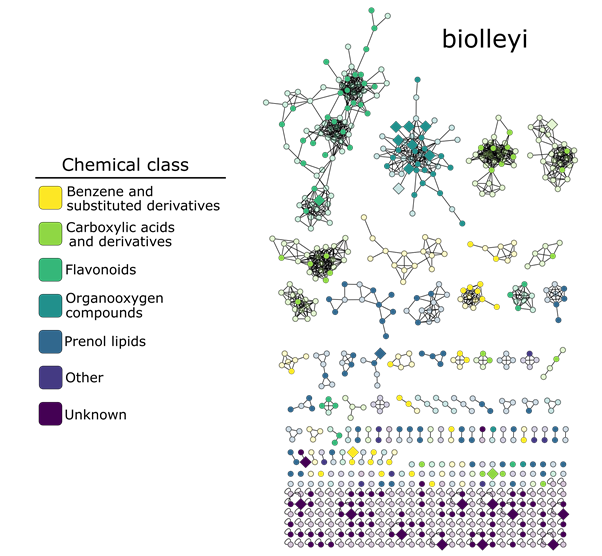


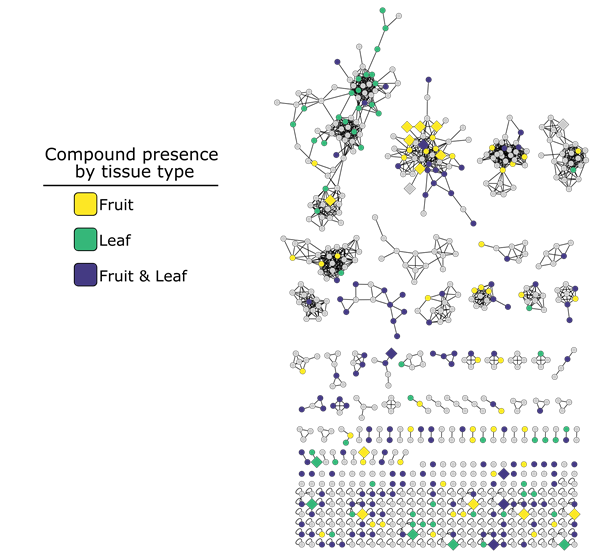


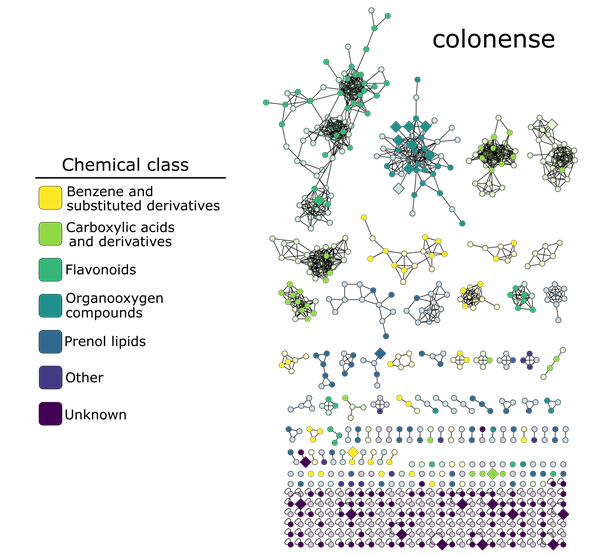


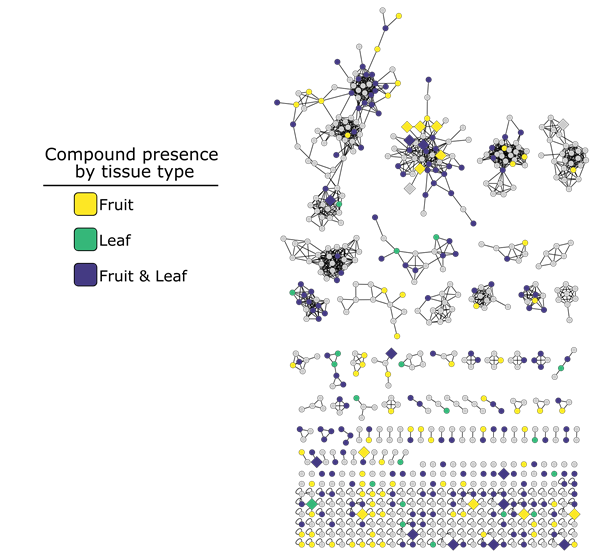


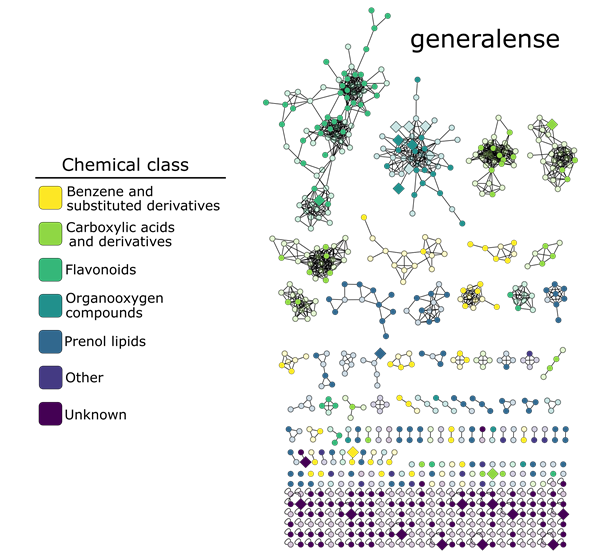


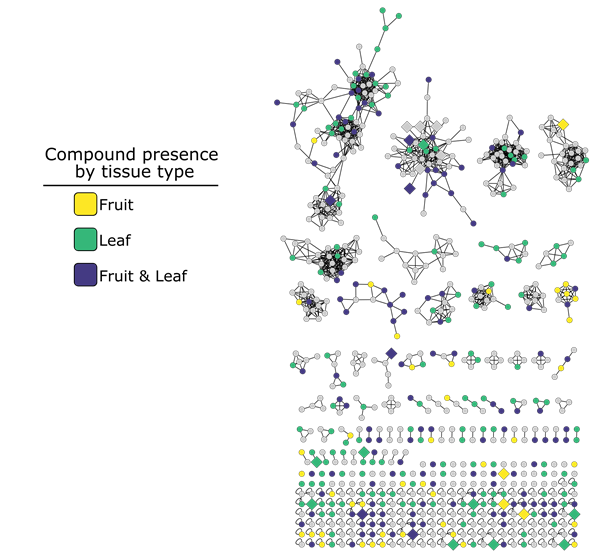


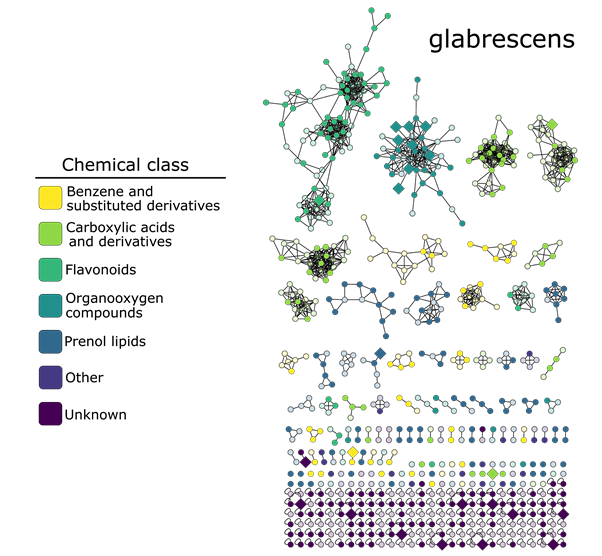


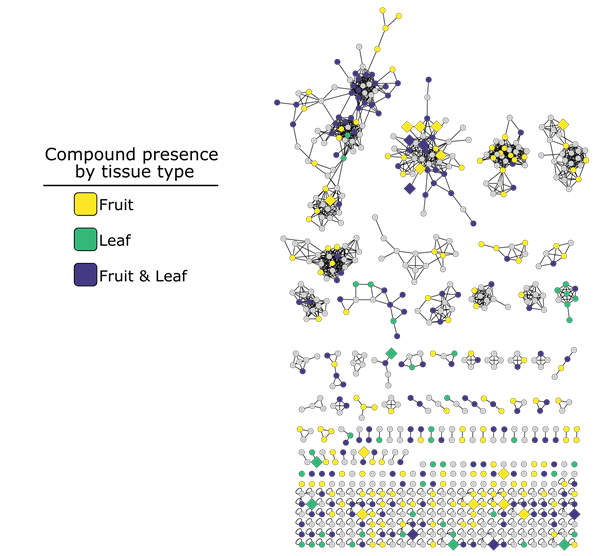


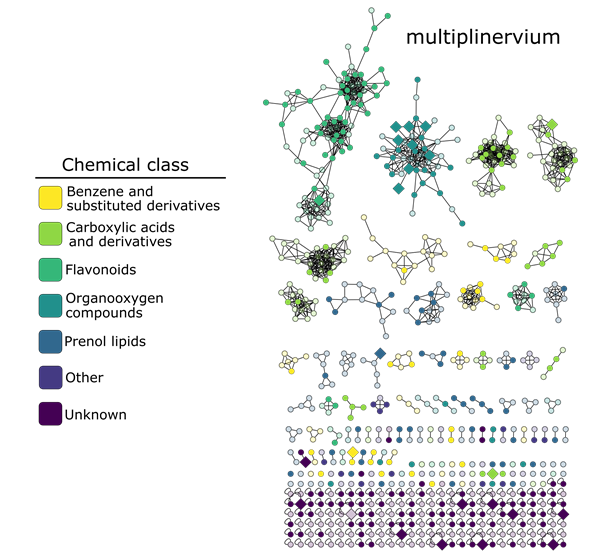


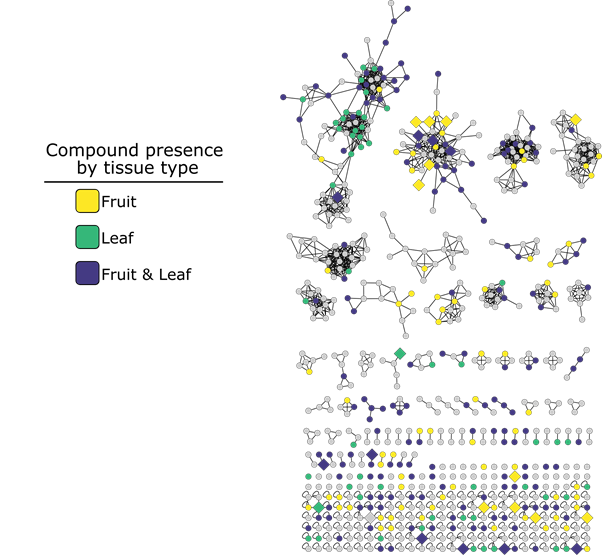


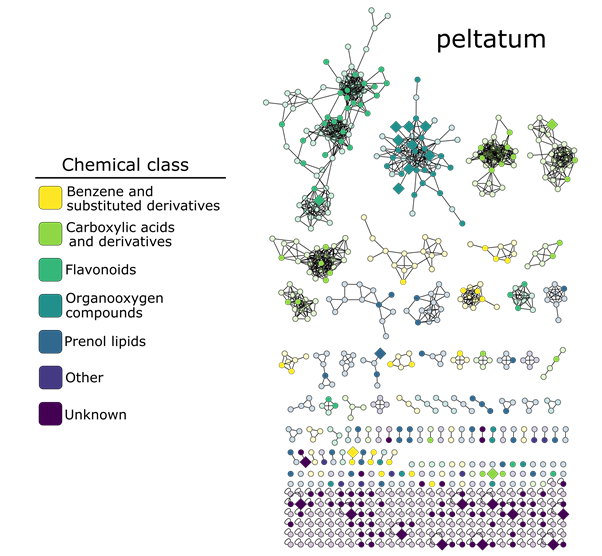


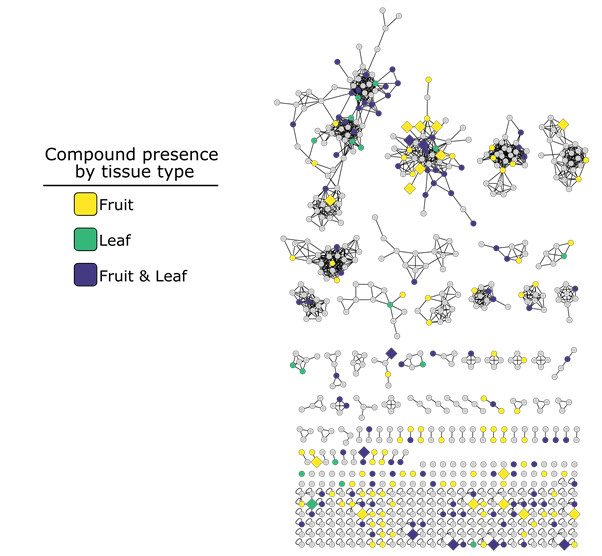


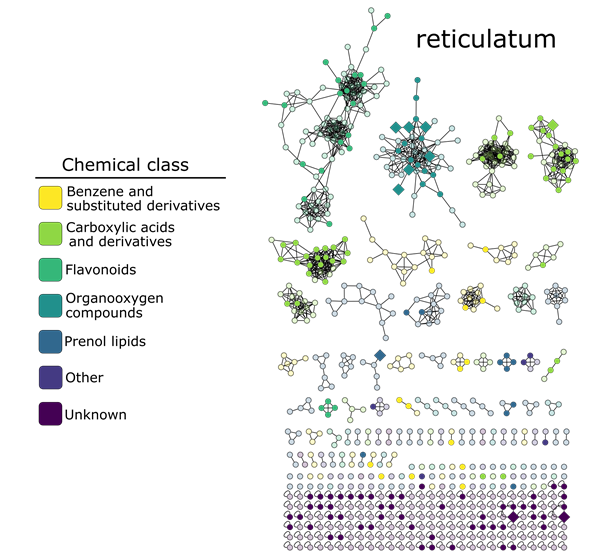


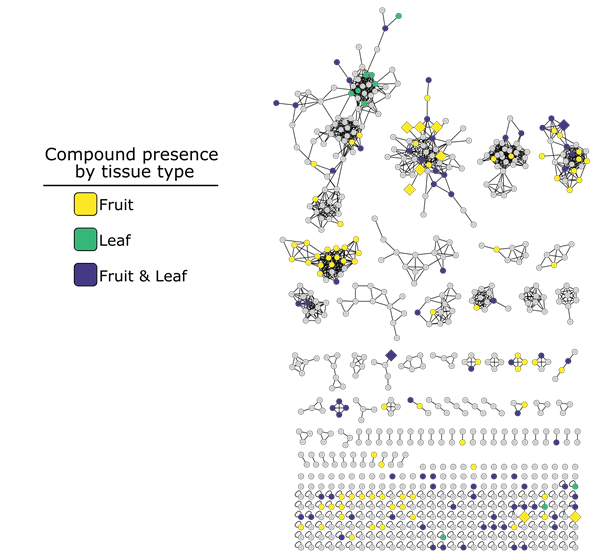


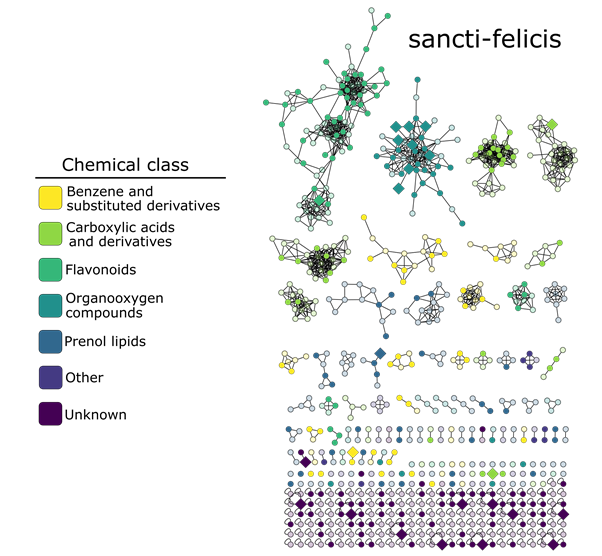


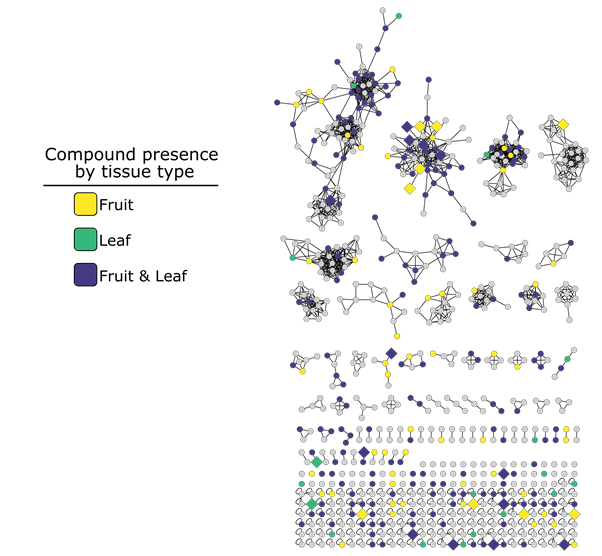


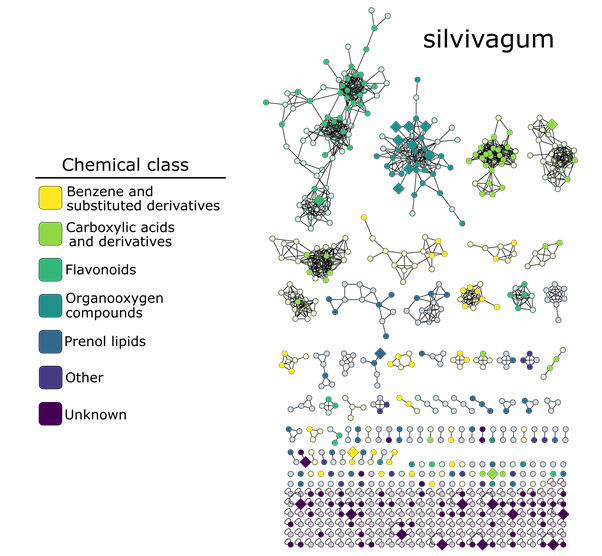


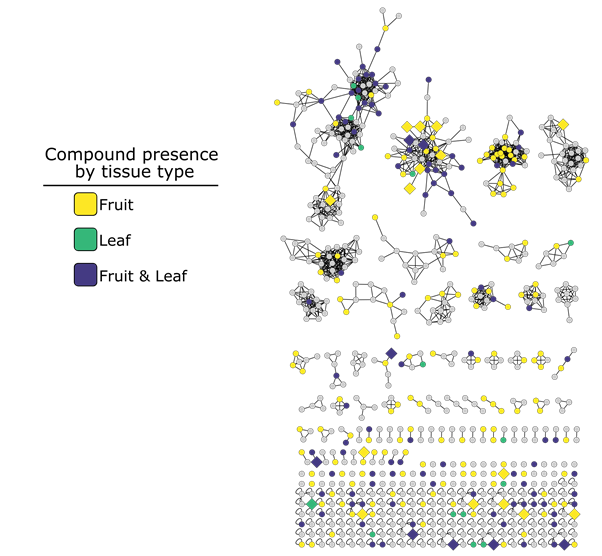


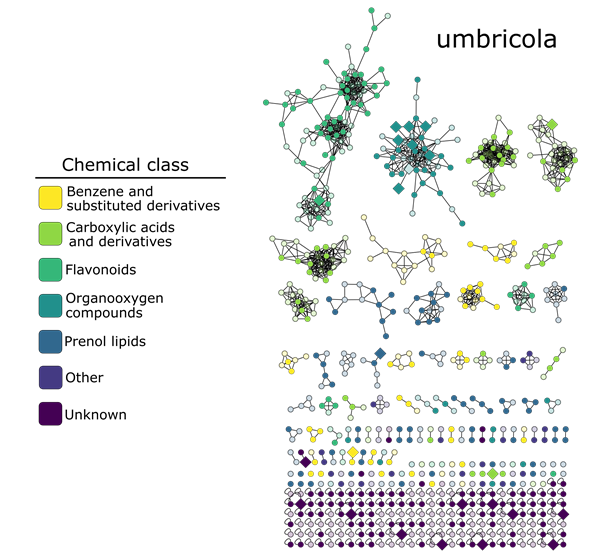


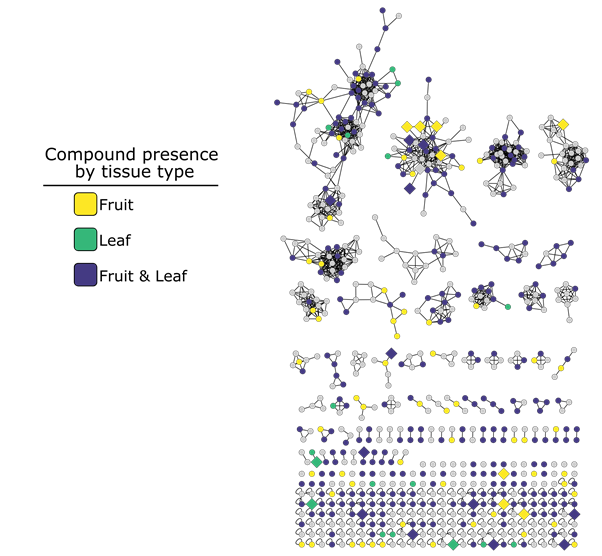

Supplement: Supplementary file 1 [file Data_Sheet_1.docx]
